# An audit of adult critical care rehabilitation processes in a UK district general hospital based on NICE guidelines

**DOI:** 10.1186/cc9952

**Published:** 2011-03-11

**Authors:** R Agarwala, N Ahmed, K Patil

**Affiliations:** 1 Croydon University Hospital, Croydon, UK

## Introduction

The majority of patients who survive critical illness have significant physical and nonphysical morbidity [1]. Current UK National Institute of Clinical Excellence guidelines set a minimum standard for the provision of rehabilitation services following ICU admission [2]. We audited local adherence to these guidelines in our 670-bed district general hospital and required a standard of 100% adherence to all parameters.

## Methods

A retrospective review of all patients ventilated, on the ICU, from September to November 2009 who survived to hospital discharge. Data reviewed included critical care and hospital discharge summaries, and notes from outreach follow-up, psychiatry follow-up and the ICU outpatient clinic.

## Results

Seventy-five patients were ventilated on our ICU during the period specified. Forty-two out of 75 (56%) survived to hospital discharge and records were available in 32 patients for inclusion in analysis. Mean total hospital stay was 44 days. Thirty-two out of 32 (100%) patients were formally assessed for their risks of developing physical and nonphysical morbidity before leaving the ICU, with short-term and medium-term rehabilitation goals being set in 28/32 (87%) of patients. Thirty-two out of 32 (100%) patients had a comprehensive clinical assessment following discharge from the ICU to identify rehabilitation needs during their ward stay. Thirteen out of 32 (41%) patients were seen by a psychologist. Seven out of 19 (37%) of those who did not see a psychologist did not meet criteria for referral, but in 10/19 (53%) who did meet criteria no referral was made. Thirteen out of 32(41%) patients were seen in an ICU outpatient clinic. Reasons given by the 59% who did not attend were multiple follow-up with other specialities and lack of perceived benefit from attendance.

## Conclusions

All patients leaving our ICU received the required standards of assessment with regards to their rehabilitation needs. A more robust system is required to ensure referral to a psychologist when indicated, as anxiety and depression following ICU admission is reported in up to 40% of patients [1]. A patient leaflet explaining these risks and the benefits of attending ICU follow-up clinics may improve outcomes.
